# Lipidomics and temporal-spatial distribution of organelle lipid

**DOI:** 10.14440/jbm.2025.0094

**Published:** 2025-01-16

**Authors:** Wenjuan Qian, Huiru Tang, Hongyan Yao

**Affiliations:** State Key Laboratory of Genetic Engineering, School of Life Sciences, Human Phenome Institute, Metabonomics and Systems Biology Laboratory at Shanghai International Centre for Molecular Phenomics, Zhongshan Hospital, Fudan University, Shanghai 200032, China

**Keywords:** Lipidomics, Organelle isolation, Organelle lipid distribution, Cellular lipid homeostasis, Inter-organelle communication

## Abstract

**Background:**

Lipids are crucial signaling molecules or cellular membrane components orchestrating biological processes. To gain insights into lipid functions and the communication between organelles, it is essential to understand the subcellular localization of individual lipids. Advancements in lipid quantification techniques, improvements in chemical and spatial resolution for detecting various lipid species, and enhancements in organelle isolation speed have allowed for profiling of the organelle lipidome, capturing its temporal-spatial distribution.

**Objective:**

This review examined approaches used to develop organelle lipidome and aimed to gain insights into cellular lipid homeostasis from an organelle perspective. In addition, this review discussed the advancements in lipid-mediated inter-organelle communication within complex physiological and pathological processes.

**Conclusion:**

With the advancement of lipidomic technologies, more detailed explorations of organelle structures and the specific lipid-mediating functions they perform are feasible.

## 1. Introduction

**Figure 1:**
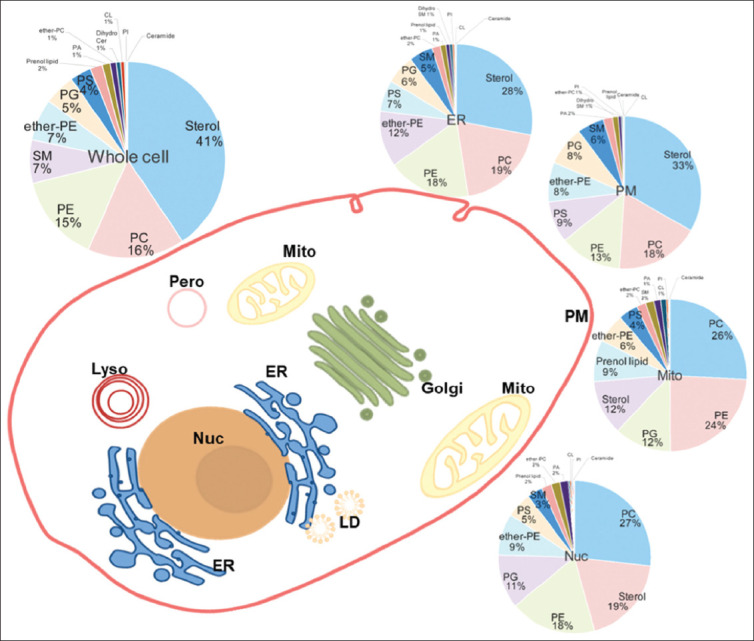
Distribution of organellar lipids in macrophages.^4^ Abbreviations: ER: Endoplasmic reticulum; Golgi: Golgi complex; LD: Lipid droplet; Lyso: Lysosome; Mito: Mitochondria; Nuc: Nucleus; Pero: Peroxisome; PM: Plasma membrane.

Lipids are fundamental building blocks of membrane structures, serving as barriers that separate the internal and external environments of eukaryotic cells. The plasma membrane (PM) segregates the interior of a cell from its extracellular space, while the inner membrane compartmentalizes organelles to facilitate distinct metabolic processes. Major organelles include the endoplasmic reticulum (ER), Golgi complex (Golgi), mitochondria (Mito), nucleus (Nuc), extracellular secretory vesicles, such as exosomes, lysosome, and the newly identified migrasome in mammalian cells.^1^-^3^ Each organelle’s membrane is composed of specific lipids that govern its functions, with their distribution patterns varying with different organelles. For instance, in macrophages, the phosphatidylcholine (PC) distribution varies significantly, with 19% in the ER and 27% in the Nuc. Similarly, sterol content ranges from 12% in the Mito to 33% in the PM (Figure 1).^4^ Recent studies on lipid-binding proteins demonstrated that membrane lipids in distinct organelles acted as signaling molecules that modulate the cellular signal transduction related to the development, survival, and effector functions of organisms.^5^-^8^ Phosphatidic acid (PA), a negatively-charged phospholipid, interacts with several important protein kinases in mammals, including Raf-1, mTOR, Akt, and members of the PKC family. These proteins are localized at the PM, ER, and Golgi, respectively. PA also binds to protein phosphatases, such as SHP-1 and PP1cγ, which are found at lipid rafts and in the nucleus, respectively, thereby mediating cellular signaling hubs.^6^,^9^-^12^

Organellar membrane lipids play a crucial role in governing inter-organelle communication within eukaryotic cells. For instance, human RdgBα1/Nir2 proteins transfer PA from the PM to the ER, while simultaneously delivering phosphoinositides (PIs) in the opposite direction.^13^,^14^ Dysfunctions in Sec14-containing proteins in humans have been linked to various diseases, including autosomal-dominant cancers and ataxia.^15^ Recent advancements in imaging techniques and whole-genome CRISPR knockout screens have discovered ether-glycerophospholipids that mediate organelle biogenesis, dynamics, and intercommunication.^16^,^17^

The comprehensive and quantitative analysis of subcellular membrane lipids is essential for studying lipid signaling and communication between organelles. Over the past 20 years, lipidomics has emerged as a field that develops comprehensive classification systems for lipids, elucidating their roles in the physiology and pathophysiology of cells and organisms.^18^-^20^ These advances in novel techniques for lipid quantification, improved chemical and spatial resolution in detecting different lipid species, and fast organelle isolation have enabled detailed profiling of the organelle lipidome, capturing its temporal-spatial distribution. Mass spectrometry (MS)-based approaches help us look into the structure of individual lipid species, providing more granular structural information about the lipid classes, including head group structures, the length of the acyl chains, and their degree of unsaturation or hydroxylation.^21^ This information contributes to a detailed, comprehensive, structural, and quantitative understanding of the molecular lipid composition of organelle membranes. The integration of microscopy and spectroscopy techniques could further reveal the dynamic distribution of organelle lipids. When combined with high-throughput MS-based lipidomics, these methods yield structural, content, and spatiotemporal data that accurately reflect the true state of organelle lipid metabolism, aiding in the research of their biological functions. Given the importance of lipid homeostasis, this review article aimed to look at current studies regarding the advantages and limitations of lipidomics and the subcellular distribution of detectable lipids. In addition, this article examined the roles of lipid-mediated inter-organelle communication in complex physiological and pathological processes within cells and organisms.

## 2. Strategies for organelle lipid analysis

The current strategies for detecting organelle lipids include isolation-detection strategy, situ detection technology, and specialized lipid probe tracing. The isolation-detection strategy involves complex procedures for the purification of the organelles, commonly employing techniques such as the density gradient ultracentrifugation, immune-isolation, and patch clamp techniques. Following isolation, lipid detection is performed using advanced equipment, including chromatography-MS and nuclear magnetic resonance technology.

### 2.1. Isolation-detection strategy for organelle lipidome

#### 2.1.1. Immunoisolation method for organelle lipidome

Immunoprecipitation-based methods have been widely developed for isolating organelles. Affinity purification is based on the principle of selecting specific proteins through highly specific interactions between antigens and antibodies. The protein characteristics of the organelle membrane surface facilitate the enrichment and purification of organelles. In this process, organelles are typically purified by binding organelle-retained proteins to fixed ligands. Commonly used naturally occurring membrane proteins include CEACAM1 and CD15 in PM,^22^,^23^ PMP70 in peroxisomes,^24^ TMEM192 and the vacuolar-type H^+^-ATPase in lysosomes,^25^,^26^ TOM20 and TOM22 in Mito,^27^,^28^ and CD63 in exosomes.^29^ However, due to the flow between organelle membranes and the use of native proteins in affinity purification, challenges arise regarding the accumulation speed and efficiency. Therefore, improving antibody specificity is essential.

Chen *et al*.^30^ developed an affinity purification method that uses the interaction between the hemagglutinin (HA) epitope and its homologous antibodies, which exhibit high affinity and specificity. With this approach, chimeric proteins containing three HA epitope tags were fused to the outer mitochondrial membrane, allowing for mitochondrial isolation in <12 min following cell homogenization.^30^,^31^ Subsequent development included the separation of HA tags from other organelles such as lysosomes, Golgi, and peroxisomes.^32^-^34^ Recently, protein purification strategies based on the fusion of streptavidin tags on target proteins, followed by affinity purification, have been employed to rapidly isolate intact lysosomes, Mito, and peroxisomes.^35^ Klemm *et al*.^36^ engineered a bait from the chimeric protein FusMidGFP, a type I transmembrane O-glycosylated raft protein, to isolate TGN-derived secretory vesicles. They introduced a high affinity 9× myc tag at the C terminus of the bait protein and inserted a TEV protease site between GFP and M9, flanked by linker regions, resulting in the immunoisolation raft carrier bait named FusMidGFPLTLM9, abbreviated as FusMidp.^36^

The success of *in vitro* organelle immunoisolation has enabled researchers to develop similar strategies that could be used for the rapid isolation of organelles *in vivo*. Bayraktar *et al*.^37^ developed MitoTag mice that express a tag consisting of 3XHA-GFP-OMP25, by utilizing an immunocapture protocol targeting the HA-tag.^37^ In addition, De Mello *et al*.^38^ developed Emx1: Cre/GFP–OMM mice, referred to as MitoTag mice, which can be used to study mitochondrial function in the brain or liver.^38^,^39^ However, the *in vivo* immunoisolation approach can be costly due to the design and breeding of genetically-modified animals. It is crucial to confirm genetic modifications using suitable methods with wild-type mice to ensure that no potential artifacts arise from these manipulations.

While immunoisolation methods could isolate pure organelles in a short time with high enrichment efficiency, contamination from adjacent organelles remains a concern. To address this problem, it is recommended to incorporate a density gradient centrifugation step when purity is an important factor in studies.^40^

#### 2.1.2. Density gradient ultracentrifugation method

The primary process for isolating organelles involves first breaking down tissue cells into a homogenate, followed by isolation of target organelles using differential ultracentrifugation and density gradient centrifugation. This process is supplemented by techniques such as Western blotting and immunofluorescence staining to assess organelle purity. Typically, the “heavy” organelles, such as crude Nuc, can be pelleted at relatively low speeds (about 500 g),^41^ while crude Mito is commonly isolated at 8000 g.^42^ In contrast, the isolation of “light” organelles, such as ER, PM, and Golgi, and organelles with active membrane vesicle activity, require multiple steps of high-speed and prolonged centrifugation.^43^,^44^ Sarmento *et al*.^45^ worked out a typical subcellular fractionation protocol applicable to tissues and cell cultures, which includes the cytoplasm, ER, extracellular vesicles, Golgi, mitochondrion-associated membrane, Mito, nucleus, PM-associated membranes, and PM.^45^ It is important to adjust specific parameters when using different media for isolating various cells or tissues.

The advantage of this traditional method lies in its ability to achieve high purity and large quantities of organelles, which is important for the accurate identification and quantification of thousands of organelle lipids. The traditional density gradient ultracentrifugation is often combined with high-throughput MS-based lipidomics to characterize the global lipid profiles of organelles. Schneiter *et al*.^46^ isolated Mito, microsomes, nuclei, peroxisomes, vacuoles, and lipid particles of *Saccharomyces cerevisiae* cells through centrifugation and analyzed the phospholipid and sterol compositions using nano-electrospray ionization tandem MS techniques. It was found that the lipid molecular profiles of the organelles were generally similar. In addition, specific lipid compositions also existed as membranes and were enriched in phosphatidylethanolamines (PEs) and phosphatidylserine (PS).^46^ Andreyev *et al*.^4^ identified 229 individual or isobaric lipid species across five organelles isolated using density gradient ultracentrifugation. This included 163 glycerophospholipids, 48 sphingolipids, 13 sterols, and five prenols. It was found that in stimulated RAW 264.7 macrophages, mitochondrial oxidized sterols increased while unsaturated cardiolipins decreased; conversely, unsaturated ether-linked PEs dropped in the ER.^4^ Although the density gradient ultracentrifugation provides highly purified organelles, it requires a relatively longer preparation time. Furthermore, the organelles isolated could still exhibit biological activities related to lipid metabolism during the isolation process, which could introduce variability that affects the accuracy of lipid quantitation.

#### 2.1.3. Patch-clamp method

A novel approach has been developed to target living organelles more efficiently by integrating patch-clamp and MS techniques. Zhu *et al*.^47^ established a single-lysosome MS platform by integrating lysosomal patch-clamp recording and nano-electrospray ionization MS (nanoESI-MS) analysis to attain concurrent metabolic and electrophysiological profiling of individual enlarged lysosomes. In this method, cells transfected to express mCherry-tagged lysosomal-associated membrane protein 1 undergo patching and membrane rupture, enabling the suction of the intra-lysosomal solution through negative pressure. The luminal constituents are then quickly transferred for MS analysis.^47^ This method ensures direct MS analysis without sample pretreatment, thereby accurately reflecting the real-time and real-situ status of a lysosome. Zhao *et al*.^48^ developed a novel technique termed in-tip solvent microextraction MS to profile PCs and triglycerides within a single lipid droplet (LD). In this study, a single LD containing a small amount of buffer solution was aspirated into a nanotip using a three-dimensional mobile manipulator. The nanotip was then backfilled with a solvent suitable for lipid extraction, followed by nanoESI-MS-based lipidomic analysis.^48^

This approach enabled the direct detection of thousands of metabolic features from a single live organelle, facilitating heterogeneity analysis. However, many of the original organelles are too small to be patched with micropipettes, necessitating their enlargement before analysis. This enlargement may alter their actual lipid characteristics. Moreover, the metabolites detected in the targeted enlarged organelles were inevitably influenced by the surrounding extracellular solution. Finally, the technique is limited in terms of throughput and automation due to the manual positioning required for each organelle.

### 2.2. Situ detection technology

The conventional “isolation-detection” strategy is suitable for metabolomic analysis of vesicular organelles; however, it inevitably causes structural damage to these organelles. Traditional lipidomic tools, such as MS, require lipid extraction before analysis, limiting their ability to detect the lipid distribution or trace lipids in live cells. Novel lipidomic strategies that use high spatial resolution MS imaging enable the exploration of complex activities within organelles. Unlike micro-pipettes, which require precise positioning and volume control, matrix-assisted laser desorption/ionization (MALDI) laser directly targets and analyzes the organelles. Phelps *et al*.^49^ established a platform for the selective analysis of single organelles using a combination of nanomanipulation workstations, microextraction techniques, and MS. They successfully sampled and detected lipids in individual LDs from adipocytes within 30 min. In addition, Castro *et al*.^50^ adapted an image-guided MALDI-MS method with high spatial resolution and throughput, facilitating metabolomic analysis of single dense-core vesicles and electron-lucent vesicles isolated from the exocrine atrial gland and red hemiduct of *Aplysia californica*, respectively.^50^

Moreover, other detection techniques, such as Raman spectroscopy, fluorescence microscopy, and fluorescence lifetime imaging microscopy, are increasingly being applied for *in situ* detection of the organelle lipidome. Plis *et al*.^51^ optimized a Raman-based lipidomic approach, called Ramanomics, which could analyze a single organelle within either live or fixed cells, thereby uncovering organelle lipid heterogeneity.^51^,^52^ Recently, the Ramanomics platform was applied to IDH1-mutated infiltrating gliomas, revealing dysregulation of phospholipid metabolism in the ER and Golgi.^53^ While Ramanomics offers spatial and temporal information about organelles, its ability to identify specific lipid species and quantify concentrations within targeted organelles requires further investigation. Consequently, this method has primarily focused on organelles with high lipid concentrations, such as LDs and ER, or has utilized Raman labels, such as deuterated^54^ or alkyne-labeled compounds,^55^ to enhance analyte detectability.

### 2.3. Real-time tracing

To investigate the inter-communications of lipids between organelles, it is essential to label and trace specific lipid species. The study of subcellular lipids has been hampered by low spatial resolutions and the inability to identify metabolites from complex mixtures without prior separation. However, significant progress has been made in these areas in recent years, particularly in imaging techniques.

Haberkant *et al*.^56^ developed a “clickable” sphingosine, known as pacSph, which serves as a precursor for the biosynthesis of other sphingolipids.^56^ PacSph contains a terminal alkyne chemical bond that can be chemically modified with fluorescent moieties to facilitate *in situ* detection through fluorescence microscopy.^56^ Feng *et al*.^57^ developed Mito-specific photoactivated sphingosines, Mito-caged sphingosine, and sphinganine, which exhibit high photo-cleavage efficiency in Mito. Using these Mito-specific photoactivated probes, the study was able to monitor sphingosine levels over time.^57^ Ancajas *et al*.^58^ described the metabolic labeling of PS in *S. cerevisiae* cells using analogs of serine, which are precursors to PS and are derivatized with azide moieties at either the amino (N-L-SerN3) or carbonyl (C-L-SerN3) groups. The experiments indicated that PS products derived from N-L-SerN3 underwent further modification to produce downstream lipids, such as PE and PC, whereas C-L-SerN3 primarily labeled PS among these lipids.^58^ Similarly, Tamura *et al*.^59^ developed a technology for the selective labeling and fluorescence imaging (microscopic or nanoscopic) of PC in organelles. By treating the cells with 500 μM N_3_-Cho for 24 h, N_3_-Cho was metabolically incorporated into cellular choline-containing phospholipids, including PC and sphingomyelin (SM). Due to its low abundance in ER-Golgi, N_3_-Cho did not label SM, ether PC, or lysoPC, thereby providing specificity for marking PC. This study provided direct evidence that the autophagosomal membrane originates from the ER through live-cell imaging.^59^ Trifunctional SM derivatives have also been utilized to visualize SM distribution and sphingomyelinase activities.^60^

In addition, expansion microscopy (ExM) provides an alternative approach for super-resolution imaging using standard fluorescence microscopes.^61^ In this technique, the targeted proteins are linked with a swellable polyelectrolyte hydrogel, which acts as a magnifying glass, achieving approximately 70 nm lateral resolution through confocal laser scanning microscopy. Götz *et al*.^61^ synthesized α-NH2-ω-N_3_-C_6_-ceramide, based on the short-chain ω-N_3_-C_6_-ceramide, that can be click-labeled with dibenzocyclooctyne-functionalized dyes for efficient labeling of cellular membranes in ExM fluorescence imaging. Confocal fluorescence images of 4× and 10× expanded cellular membranes demonstrated that sphingolipid ExM labeling was dense enough to support nanoscale resolution imaging of continuous membrane structures and thin membrane protrusions. Furthermore, when integrated with structured illumination microscopy, sphingolipid ExM achieved a 10 – 20 nm spatial resolution, approaching those of electron microscopy.^61^

### 2.4. Other methods

Peter *et al*.^62^ developed a versatile method dubbed Mass tagging-Enabled TrAcking of Lipids In Cells to track inter-organelle lipid flux within cells. This technique uses cyclopropane fatty-acyl phospholipid synthase, an enzyme that introduces a methylene group at double bonds into phospholipid fatty acyl chains, forming a cyclopropane ring and a +14 Da mass shift. In a classical approach, Ardail *et al*.^63^ used isotopic lipids to trace lipids in the PM and Mito. The study pre-labeled cells with [3H]-palmitate for 24 h and observed a peak of [^3^H]-ceramide in lipid rafts 15 min after irradiation, correlating with a decrease in the [^3^H]-SM content within those rafts.^63^

## 3. Organelle distribution of specific lipids

### 3.1. Organelle distribution of cholesterol

Cholesterol is an essential lipid constituent of cell membranes and functions as a precursor for oxysterols, bile acids, steroid hormones, and vitamin D.^64^ It is a hydrophobic ringed lipid molecule characterized by four fused hydrocarbon rings with a polar hydroxyl group at the one end and an eight-carbon branched aliphatic tail at the other end. The structure is rigid and predominantly apolar, with the small hydroxyl group being the only polar group in the molecule. This unique architecture allows cholesterol to integrate seamlessly into the membrane lipid bilayer. Cholesterol exerts multiple effects on membrane lipid bilayers, including alterations in fluidity, thickness, and curvature.^65^ It is heterogeneously distributed among intracellular membranes to perform various biological functions.^64^ It is generally believed that cholesterol concentrations increase along the biosynthetic secretory pathway.^66^ The ER contains minimal cholesterol, while endosomes and PM exhibit relatively high levels. The Golgi has an intermediate cholesterol content that increases from the cis to the trans side.^67^

Current studies revealed slight variations in the organelle distribution of cholesterol. Optical observations indicated the following distribution of cholesterol content: endosome > PM > lysosome > Golgi > ER.^68^ In analyses of macrophage organelles, the distribution was found to be: PM > ER > Mito > Nuc.^4^ Conversely, in lipidomic analyses of mouse stromal tumor cells, the distribution was reported as: PM > Mito > ER.^69^ These findings suggest that there may be differences in the cholesterol content between ER and Mito in mouse stromal tumor cells compared to macrophages (Table 1).

**Table 1:** Abundance, location, and measurement of cholesterol, diacylglycerol, phosphatidylserine, and sphingomyelin in eukaryotic cells

Lipids	Organelle distribution	Analysis method	Cells	References
Cholesterol	Endosome > PM > Lyso > Golgi > ER	Optical observation	Primary human fibroblasts	^68^
	PM > ER > Mito > N	LC-MS	Macrophage	^4^
	PM > Mito > ER	LC-MS	Mouse stromal tumor cell	^69^

DAG	*sn*-1,2 DAG: ER > Mito > PM > LD; *sn*-2,3 DAG: LD > ER > Mito > PM; *sn*-1,3 DAG: LD > Mito > ER > PM	LC-MS	Rat liver	^70^
	*sn*-1,2 DAG: Mito > ER > LD > PM; *sn*-2,3 DAG: Mito > LD > ER > PM; *sn*-1,3 DAG: Mito > LD > ER > PM	LC-MS	Human liver	^70^
	Total DAG: LD > ER > Mito > PM; *sn*-1,2 DAG: LD > ER > Mito > PM; *sn*-2,3 DAG: LD > ER > PM≈Mito; *sn*-1,3 DAG: LD > ER > Mito > PM	LC-MS	White adipose	^71^
	*sn*-1,2 DAG: ER > PM > Mito > LD; *sn*-1,3 DAG: Mito≈PM > ER > LD; *sn*-2,3 DAG: PM≈Mito > LD > ER	LC-MS	Mice gastrocnemius	^72^

PS	PM > endosome > Golgi > ER	Optical observation	A431 cell	^73^
	PM > ER > Mitochondria	Optical observation	Liver	^73^

SM	PM > PM associated membrane > ER > Mito > Mito associated membrane	LC-MS	MA-10 mouse Leydig tumor cell	^69^
	PM > ER > nucleus > Mito > dense microsome	LC-MS	Macrophage	^4^
	PM > lysosomes > Golgi > ER	TCL	BHK fibroblasts	^74^

Abbreviations: BHK: Baby hamster kidney; DAG: Diacylglycerol; ER: Endoplasmic reticulum; Golgi: Golgi apparatus; LC-MS: Liquid chromatography-mass spectrometry; LD: Lipid droplet; Lyso: Lysosome; Mito: Mitochondria; Nuc: Nucleus; PM: Plasma membrane; PS: Phosphatidylserine; SM: Sphingomyelin; TCL: Thin layer chromatography.

### 3.2. Organelle distribution of diacylglycerol (DAG)

DAG is a simple lipid composed of a glycerol molecule linked through ester bonds to two fatty acids at positions 1 and 2. DAG exists in three different isomeric forms: *sn*-1,2, *sn*-2,3, and *rac*-1,3 DAG.^75^ Various enzymes, including transferases, kinases, and lipases, can discriminate between these DAG isomers. In addition, proteins that interact with DAG are localized in different subcellular compartments.^76^,^77^ This localization suggested that distinct DAG isomers may play different roles in cell signaling. Of note, only *sn*-1,2 DAG can bind to the C1 domain of signaling effectors, which is a structural module involved in various signaling pathways.^75^ Hence, the generation and quantification of *sn*-1,2 DAG have been extensively investigated. At the PM, *sn*-1,2 DAGs are primarily generated from the breakdown of phosphorylated phosphatidylinositol lipids (PIPs) by PI-specific phospholipase C. In the ER and Golgi, *sn*-1,2 DAGs can be produced from triacylglycerols (TAGs) through TAG lipase, PA through PA phosphatase, PC through sphingomyelin synthase, or monoacylglycerols through monoacylglycerol-acyltransferase.^78^ Several studies have revealed that the inhibition of the *sn*-1,2-DAG-protein kinase C epsilon type (PKCε) signaling axis may be a potential strategy for treating metabolic diseases, such as hepatic insulin resistance and type 2 diabetes.

PM-bound *sn*-1,2 DAG contributes to hepatic insulin resistance by activating PKCε.^70^ In one study, liver tissues were separated into ER, Mito, PM, LD, and cytosol using differential ultracentrifugation to measure the DAG stereoisomer content.^70^ In wide-type rat liver, the distribution of *sn*-1,2 DAG was ER > Mito > PM > LD. Conversely, the distribution of *sn*-2,3 DAG was LD > ER > Mito > PM, and the distribution of *sn*-1,3 DAG was LD > Mito > ER > PM. In the liver of human individuals who were insulin sensitive, the concentration of *sn*-1,2 DAG was Mito > ER > LD > PM, while the concentration of *sn*-2,3 DAG was Mito > LD > ER > PM, and the concentration of *sn*-1,3 DAG was Mito > LD > ER > PM. In another study of white adipose tissue, the total DAG proportion was LD > ER > Mito > PM, the proportion of *sn*-1,2 DAG was LD > ER > Mito > PM, the proportion of *sn*-2,3 DAG was LD > ER > PM≈Mito, and the proportion of *sn*-1,3 DAG was LD > ER > Mito > PM.^71^ Another study on mice gastrocnemius demonstrated the concentration of *sn*-1,2 DAG to be highest in ER > PM > Mito > LD, the concentration of *sn*-1,3 DAG was Mito≈PM > ER > LD, and the concentration of *sn*-2,3 DAG was PM≈Mito > LD > ER.^72^

Abulizi *et al*. studied young and aged liver-specific MTTP knockout mice to investigate the development of lipid-induced hepatic insulin resistance.^79^ Their results showed that in young mice hepatic cells, the concentration of *sn*-1,2 DAG was PM > LD > ER > Mito, the concentration of *sn*-1,3 DAG was LD > ER > PM > Mito, and the concentration of *sn*-2,3 DAG was LD > ER > PM≈Mito. In aged mice, the distribution of DAG isomers changed. The concentration of *sn*-1,2 DAG was Mito > LD > ER > PM, the concentration of *sn*-1,3 DAG was LD > ER≈Mito > PM, and the concentration of *sn*-2,3 DAG was LD > ER≈Mito > PM.^79^ Zheng *et al*.^78^ found that phytochemical atractylenolide II (AT II) improved obesity-induced insulin resistance through the *sn*-1,2-DGK/PKCε signaling axis.^80^ In HL-7702 cells treated with PA, relative *sn*-1,2 DAG content was PM > Mito > ER > LD, and AT II significantly decreased the *sn*-1,2-DAG contents of ER, Mito, and PM. These studies on the quantitative distribution of DAG isomers within individual organelles provide important insights into their functional roles across organelles. The organelle distribution of DAG is summarized in Table 1.

### 3.3. Organelle distribution of phosphatidylserine and PI

Phosphatidylserine plays a central role in cell signaling and the biosynthesis of other lipids. The electrostatics of the membrane are significantly influenced by PS concentrations, which introduce a negatively-charged phosphate group attached to the amino acid serine at the hydroxyl end.^81^ Measurements of subcellular electrostatics suggest that PS is concentrated along membranes of the secretory pathway, with the following hierarchy: PM > Golgi > ER.^67^ The function of PS is determined by its concentration and orientation within the membranes. However, the exact concentration, subcellular distribution, and transmembrane topology of this crucial phospholipid remain unclear. Fairn *et al*.^80^ used cells transfected with a GFP-tagged C2 domain of lactadherin to detect PS exposed on the cytosolic leaflet of the plasmalemma and organelle membranes using light and electron microscopy.^73^ The results revealed the relative distribution of PS in subcellular fractions, showing that PS was enriched in the following order: PM > endosome > Golgi > ER. In addition, organelles isolated from the liver tissue exhibited PS concentrations in the order of PM > ER > Mito.^73^ These results indicated that PS in the lumenal monolayer of the ER and Golgi complex becomes cytosolically exposed at the trans-Golgi network, suggesting that transmembrane flipping of PS might contribute to cargo exit from the Golgi complex.

Inositol phospholipids are known to play important regulatory roles in cell physiology, including signal transduction at the membrane surface, regulation of membrane traffic, nuclear events, and membrane permeability and transport functions.^82^ Reversible phosphorylation of the inositol ring at positions 3, 4, and 5 resulted in the generation of seven PI derivatives. Each of these PI has a unique subcellular distribution and is predominantly localized in specific membrane subsets, making them valuable markers of different organelles^83^,^84^ (Table 2).^85^ For example, PI(5)P binds to TOM1 located in late endosomes or multivesicular bodies.^86^ PI(4,5)P_2_ was concentrated in at the PM but can also be detected in other subcellular compartments. PI(3,4,5)P_3_ is possibly enriched in raft-like structures, while PI(3,4)P_2_ is mostly found in the PM and early endocytic pathways. PI(4)P is enriched in the Golgi but is also present in the PM. PI(3)P is concentrated in early endosomes, whereas PI(3,5)P_2_ is found in late compartments of the endosomal pathway.^87^ The subcellular distribution of PIs has been extensively reviewed^85^ and has been summarized in Table 2. With advancements in lipidomics, it is expected that the distribution of PIs and their dynamic changes in organelles will be comprehensively studied.

**Table 2:** Abundance, location, and measurement of phosphoinositide lipids in eukaryotic cells^85^

Lipid	Abundance	Distribution	Assays for measurement
Phosphatidylinositol (a.k.a. PI, PtdIns)	∼80 mol% of total cellular PPIs	Abundant in ER but potentially all membranes	TLC, HPLC-MS/MS, [^3^H] or [^32^P] radiolabeling to equilibrium

Phosphatidylinositol 4-phosphate (a.k.a. PI (4) P, PtdIns4P, PI4P)	∼2 – 5 mol% of total cellular PPI	PM, endosomes, trans-Golgi	TLC, HPLC-MS/MS, [^3^H] or [^32^P] radiolabeling to equilibrium, immunofluorescence, fluorescent biosensor (P^4^M)

Phosphatidylinositol 3-phosphate (a.k.a. PI (3) P, PtdIns3P, PI3P)	∼0.2 – 0.5 mol % of total cellular PPI	Early endosomes	TLC, [^3^H] or [^32^P] radiolabeling to equilibrium, fluorescent biosensors (FYVE)

Phosphatidylinositol 5-phosphate (a.k.a. PI (5) P, PtdIns5P, PI5P)	∼0.01 mol% of total cellular PPI	PM, endosomes, nuclear envelope	TLC, [^3^H] or [^32^P] radiolabeling to equilibrium

Phosphatidylinositol 4,5-bisphosphate (a.k.a. PI (4,5) P_2_, PtdIns4,5P_2_, PI4,5P_2_, PIP_2_)	2 – 5 mol% of total PPI	PM, recycling endosomes, lysosomes	TLC, HPLC-MS/MS, [^3^H] or [^32^P] radiolabeling to equilibrium, immunofluorescence, genetically encoded fluorescent biosensor (PH domain from PLCδ^1^ for PM labeling), ion channel currents.

Phosphatidylinositol 3,4-bisphosphate (a.k.a. PI (3,4) P_2_, PtdIns3,4P_2_, PI3,4P_2_)	< 0.1mol% of total PPI	PM, early endosomes	TLC, HPLC-MS/MS, [^3^H] or [^32^P] radiolabeling to equilibrium, immunofluorescence, genetically encoded fluorescent biosensor (TAPP^1^ PH domain)

Phosphatidylinositol 3,5-bisphosphate (a.k.a. PI (3,5) P_2_, PtdIns3,5P_2_, PI3,5P_2_)	<∼2 mol% of total PPI	Late endosomes and lysosomes	TLC, HPLC-MS/MS, [^3^H] or [^32^P] radiolabeling to equilibrium, immunofluorescence, genetically encoded fluorescent biosensor (FYVE)

Phosphatidylinositol 3,4,5-trisphosphate (a.k.a. PI (3,4,5) P_3_, PtdIns3,4,5P_3_, PI3,4,5P_3_, PIP_3_)	< 0.05% of total PPI	PM, some endocytic compartments	TLC, HPLC-MS/MS, [^3^H] or [^32^P] radiolabeling to equilibrium, immunofluorescence, genetically encoded fluorescent biosensors (AKT, BTK biosensors).

Abbreviations: AKT: Protein kinase B; BTK: Bruton tyrosine kinase; ER: Endoplasmic reticulum; FYVE: Fab1, YOTB/ZK632.12, Vac1, and EEA1; HPLC-MS/MS: High-performance liquid chromatography-tandem mass spectrometry; P4M: PtdIns4P binding of SidM; PH: Pleckstrin homology; PM: Plasma membrane: PPI: Polyphosphoinositide; TAPPI: Phosphoinositol 3,4-bisphosphate-binding protein; TLC: Thin-layer chromatography.

### 3.4. Organelle distribution of sphingolipids

Sphingolipids are built on a sphingosine backbone, which is linked to fatty acids via amide bonds to form ceramides. The sphingoid base is synthesized from a long-chain fatty acyl-CoA and serine.^88^ Ceramide is the simplest sphingolipid, consisting solely of hydrogen and an amide-linked fatty acid. The metabolic pathways of sphingolipids involve various enzymes located in specific subcellular organelles. Sphingolipids can be synthesized primarily through the *de novo* pathway in the ER and degraded through the salvage pathway, mainly in lysosomes. In addition, other organelles, including the PM, Golgi, Mito, and Nuc, also contribute to metabolic homeostasis.^89^

In MA-10 mouse Leydig tumor cells, the concentration of SM was found to be highest in PM, followed by PM-associated membrane, ER, Mito, and Mito-associated membrane.^69^ Ceramide distribution was observed to be PM > ER > PM-associated membrane > Mito-associated membrane > Mito. Allan *et al*.^89^ summarized lipid distribution in the membranes of Baby Hamster Kidney (BHK) cells based on published information, reporting the distribution of sphingomyelin as follows: PM > lysosomes > Golgi > ER in the BHK fibroblasts.^74^ In macrophages, the concentration of sphingolipids was PM > ER > Nuc > Mito > dense microsome.^4^ As more organelle data from various cell types or organisms become available, the generality and differences in sphingolipid distribution will be disclosed, which is crucial for the understanding of their functional roles and lipid communication. The subcellular distribution of SM lipids exhibits complexity across different cell types. It can be concluded, from these studies, that the content of SM was higher in PM than in ER, likely due to its significant role in membrane structure formation. This distribution pattern aligns with that of PS, indicating a co-sorting relationship between SM in the outer leaflet and PS in the inner leaflet of membranes.^90^ The organelle distribution of sphingolipids is summarized in Table 1.

## 4. Lipids mediate organelle biogenesis and communication

The diverse distribution of lipids across organelles is closely related to organelle communications, encompassing such processes as transport, breakdown, recycling, synthesis, or storage of organellar lipids. Advances in lipidomics have enabled comprehensive studies of the intricate network of inter-organelle interactions. Following organelle isolation, the nuclear and mitochondrial lipidomes in mouse liver were characterized, revealing diurnal oscillations of lipid accumulation. Notably, organellar PC and PE derivatives exhibited similar temporal accumulation profiles in both compartments.^91^ This study provided insights into the potential temporal and spatial connections between lipid species within organelles.

A multitude of techniques and approaches is needed to gain a broader understanding of how lipids mediate organelle communication. Three-dimensional (3D) imaging techniques can visualize organelle contact sites as well as the topology and morphology of various organelles.^92^ A recent study employed a 3D imaging technique in combination with a whole-genome CRISPR knockout screen and illustrated that disruptions in mitochondrial function triggered cell-wide changes in the structure and function of other organelles, including peroxisomes, Golgi, and ER.^17^ Further lipidomic analyses identified that glycerophospholipid metabolism in ER, Mito, Golgi, and peroxisomes regulated organelle biogenesis and membrane remodeling through membrane contact sites and lipid-transfer mechanisms. The homeostasis of glycerophospholipid involves an intricate network of communication among Mito, peroxisomes, ER, and the Golgi. Remarkably, ether-glycerophospholipids were discovered to play crucial roles in modulating organelle biogenesis, dynamics, and intercommunication.^16^,^17^ A multi-spectral organelle imaging approach was applied to track lipid flow,^93^ demonstrating interactions among six key lipid metabolic organelles, including LDs, ER, Mito, Peroxisomes, Golgi, and lysosomes, and highlighting the pivotal function of LDs in regulating inflammation.^93^

Lipids are essential for mediating inter-organelle communication. The crosstalk between the ER and Mito, Mito and lysosomes, Mito and Nuc, lysosomes and Nuc, as well as peroxisome biogenesis from the ER and Mito has been extensively reviewed.^16^,^94^,^95^ In particular, cholesterol is transported between Mito, lysosomes, peroxisomes, ER, and the Nuc through membrane contact sites. Ether-phospholipids and plasmalogens play important roles in facilitating communication between the peroxisomes and both the ER and Mito. The PC, PA, PE, and PI are involved in signaling networks among Nuc, ER, and Mito.^94^

## 5. Organelle lipid composition affects cancer metastasis

Lipids play crucial roles in a wide array of physiological and pathological processes, especially in cancer metastasis. Changes in organellar membrane lipids within cancer cells can promote malignant progression. During cancer metastasis, significant alterations in cell morphology, cellular motility, and cell polarity were observed,^96^-^98^ and were closely related to the modification and regulation of organelle lipids. Cholesterol and SM, the primary lipids in the PM, form the “lipid rafts,” to further regulate cell adhesion and migration. Low membrane cholesterol levels promote cancer metastasis, while high levels of SM have been found to be associated with accelerated cancer migration and invasion.^96^,^97^ Glycolipids, a major class of sphingolipids, are predominantly located in the outer leaflets of PMs and are enriched in the cerebroside, GM2, GD2, and GD3. These glycolipids promote the migration, proliferation, and adhesion of tumor cells.^98^-^100^ Sphingolipid 1-phosphate (S1P), a tumor-promoting factor, may attenuate autophagy by regulating the SPHK1-TRAF2-BECN1-CDH1 signaling cascade in lysosomes, thereby influencing cell fate. S1P has also been used to treat cell resistance and metastasis.^101^ In addition, tumor cell-derived SM and S1P are enriched in exosomes, facilitating the communication between tumor cells and promoting cancer angiogenesis, invasion, metastasis, and drug resistance.^102^-^106^ Sphingolipids can be synthesized or recruited into Mito. A decrease in SM and ceramide levels can affect membrane stiffness, leading to increased oxidative damage and disrupted mitochondrial functions in cancer cells.^107^ Mitochondrial carnitine has been shown to mitigate TGF-β1-induced cellular epithelial-mesenchymal transition and reduce the migration ability of retinal pigment epithelial cells by inhibiting the Erk1/2 and JNK pathways while upregulating the expression of peroxisome proliferator-activated receptor γ.^108^

**Figure 2:**
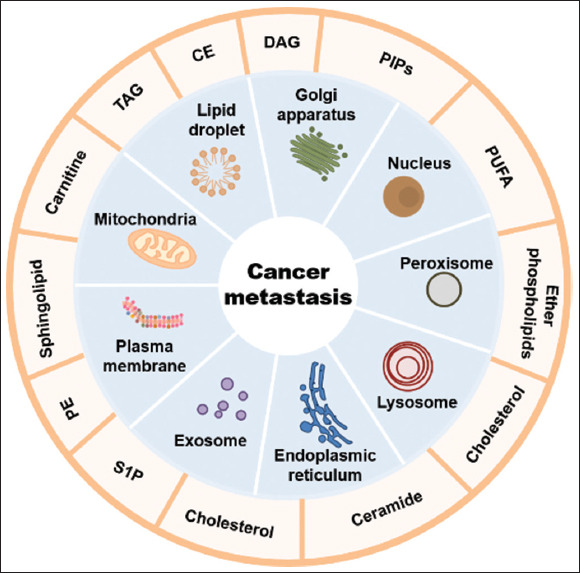
The lipid distribution of organelles in cancer metastasis. An overview highlighting some of the organelles that are involved in lipid metabolism during cancer metastasis (inner ring) and organellar lipids that may specifically enrich (outer ring). Abbreviations: CE: Cholesteryl ester; DAG: Diacylglycerol; PE: Phosphatidylethanolamine; PIP: Phosphoinositide; PUFA: Polyunsaturated fatty acid; S1P: Sphingosine-1-phosphate; TAG: Triacylglycerol.

An increasing number of lipids were found to play crucial roles in the metastasis of cancer cells. For instance, PI(4)P is enriched in the trans-Golgi network and recruits GOLPH3 to direct vesicle trafficking, thereby driving cancer metastasis.^109^-^111^ DAG in Golgi enhances shearing and affects cell polarization through the lipin-1/DAG/Arf1/SREBP pathway, contributing to cancer metastasis.^112^ PIPs and polyunsaturated fatty acids within the Nuc can bind to nuclear receptors and play significant roles in regulating cell metastasis.^113^ The metabolism of ether phospholipids in peroxisomes,^114^ *de novo* synthesis and salvage pathways of ceramides and cholesterol in the ER and lysosomes,^115^ as well as the homeostasis of TAGs and CEs in LDs^116^ are all associated with the metastasis of cancer cells (Figure 2). Recent studies on lipid roles have also led to the development of a series of anticancer drugs targeting lipid pathways (Table 3).

**Table 3:** A summary of anticancer drugs targeting lipid metabolism^117-119^

Target	Lipids	Drug	Type of cancer	Phase of development
SREBPs	Cholesterol	Sibilinin	Breast, head and neck, lung and prostate cancer	Preclinical
		Betulin	Hepatocellular carcinoma	
		Fatostatin	Glioblastoma, osteosarcoma, breast, prostate cancer	

HMGCR	Cholesterol	Statin	Many cancers	Approved
	Simvastatin	Prostate cancer	Preclinical

ACAT1	Cholesterol	ATR-101	Many cancers	Approved
		Avasimibe		

PLD	Phospholipids	VU0359595	Breast cancer	Preclinical
		VU-0155069	Breast cancer	

S1P	Sphingosine-1-phosphate	FTY720	Lung cancer	Preclinical
		JTE013	Bladder cancer	
		AB1	Neuroblastoma	
	Sphingomab	Renal cell cancer	Phase II	

SPHK	Sphingosine/Ceramide	SK1-I	Glioblastoma multiform	Preclinical
		PF543	Colorectal cancer	
	ABC294640	Advanced solid tumors	Phase Ib and II	

Ceramide	Ceramide/SM	LCL-521	Prostate cancer	Preclinical
		LCL-385		

CPT1	Carnitine	Etomoxir	Prostate cancer, breast cancer, glioblastoma	Preclinical
		Ranolazine	Prostate cancer	
		Perhexiline	Breast cancer, prostate cancer, lymphocytic leukemia	

GPX4	Lipid desaturation	RSL3	Ovarian adenocarcinoma cells	Preclinical

FASN	FA	C75	Breast cancer, glioblastoma multiform, renal cell cancer, mesothelioma, glioma, lung cancer, melanomas	Preclinical
		Cerulenin	Ovarian cancer and breast cancer	
		Orlistat	Prostate cancer, melanoma, glioma endometrial cancer, melanomas, oral tongue squamous cell carcinoma	
		Triclosan	Prostate cancer, breast cancer	
		Amentoflavone	Breast cancer	
		EGCG	Lung cancer, breast cancer	
		TVB-3166	Ovarian cancer	

CD36	FA	ABT-510	Glioblastoma, melanoma, renal cell carcinoma	Phase I

Abbreviations: ACATI: Acetyl-CoA acetyltransferase 1; CPT1: Carnitine palmitoyltransferase I; EGCG: Epigallocatechin gallate; FA: Fatty acid; FASN: Fatty acid synthase; GPX4: Glutathione peroxidase 4; HMGCR: 3-hydroxy-3-methyl-glutaryl-coenzyme A reductase; PLD: Phospholipase D; RSL3: RAS-selective lethal; S1P: Sphingolipid 1-phosphate; SM: Sphingomyelin; SPHK: Sphingosine kinase; SREBP: Sterol regulatory-element binding proteins.

## 6. Conclusion

Lipids confer specific structure and signaling roles to organelles. Understanding the relationship between lipids and individual organelle structures, as well as their involvement in signaling transduction, is essential for unraveling the specificity and functions of organelle structures. Lipidomics provides the techniques for these studies, making comprehensive and quantitative analysis of organelle lipids crucial. The distribution of lipids within organelles reveals both common and specific characteristics, and further measurements of lipid distribution at nanometer resolution, combined with 3D visualization, will enhance our understanding of how lipids contribute to the formation of specific intra-organelle structures, such as cristae of Mito and bulges of the ER. With the advancement of lipidomic approaches, more detailed explorations of organelle structures and the specific lipid-mediating functions they perform can be expected.

A comprehensive analysis of the organellar lipid crosstalk and subcellular lipid-protein interactions can help develop and verify hypotheses regarding underlying lipid transfer mechanisms and regulatory principles. Whole-genome CRISPR knockout screens are ideal for elucidating lipid networks and lipid-mediated organelle communication. By combining subcellular proteomics with lipidomics, researchers can identify common lipid-protein complexes across different cell types. Precisely controlling organelle lipid content through lipid transporters or metabolic enzymes, along with subtle modifications in lipid chain length or unsaturation, may be critical not only for understanding the functions of the organelles but also for developing better therapies to treat cancers, neurodegeneration, aging, and other metabolic diseases.

## Data Availability

Not applicable.
